# Aging-associated immune signature as a predictor of mortality in end-stage renal disease: results from the longitudinal iESRD study

**DOI:** 10.1186/s12979-025-00554-4

**Published:** 2025-12-29

**Authors:** Kai-Hsiang Shu, TienYu Owen Yang, Graham Pawelec, Feng-Jung Yang, Wan-Chuan Tsai, Yu-Sen Peng, Shih-Ping Hsu, Yi-Fang Chuang, Yen-Ling Chiu

**Affiliations:** 1 Nephrology Division, Department of Medicine, Far Eastern Memorial Hospital, New Taipei City, Taiwan; 2 Graduate Institute of Immunology, College of Medicine, National Taiwan University, Taipei, Taiwan; 3 Nuffield Department of Population Health, University of Oxford, Oxford, UK; 4 Institute of Immunology, University of Tübingen, Tübingen, Germany; 5 School of Medicine, College of Medicine, National Taiwan University, Taipei, Taiwan; 6 Department of Internal Medicine, National Taiwan University Hospital, Taipei, Taiwan; 7 Department of Medical Genetics, National Taiwan University Hospital, Taipei, Taiwan; 8 Institute of Public Health, School of Medicine, National Yang Ming Chiao Tung University, Taipei, Taiwan; 9 Graduate Institute of Clinical Medicine, College of Medicine, National Taiwan University, Taipei, Taiwan; 10 Graduate Institute of Medicine, Yuan Ze University, Taoyuan, Taiwan

**Keywords:** Lymphocyte, Monocyte, Principal component analysis, Mortality, Hemodialysis

## Abstract

**Background:**

Accelerated immune aging has been implicated in patients with end-stage kidney disease, but a detailed examination of immune profiles correlated with long-term outcomes for these individuals has never been performed. Therefore, we conducted a prospective observational study (“Immunity in end-stage renal disease”, iESRD) to investigate the effects of immune aging on mortality among these patients. An exploratory panel of immune cell subsets was analyzed by flow cytometry at baseline (neutrophils, CD3-negative lymphocytes, CD4 and CD8 T cell differentiation stages, and three subsets of monocytes). Immune cell distribution patterns were identified through data-driven principal component analysis (PCA).

**Results:**

A total of 409 hemodialysis patients (mean age 61.7 years, range 29.5–89.1) were enrolled and followed for three years, during which 75 deaths occurred. Compared with survivors, deceased patients displayed features of more advanced immune aging, which was also correlated with older chronological ages. For individual subset, a higher level of CD8 naïve cells and a lower level of CD4 effector memory cells at baseline were associated with lower mortality. For comprehensive immune signature, principal component analysis identified three major patterns, with PC3—characterized by loss of naïve T cells and enrichment of effector memory T cells and non-classical monocytes—strongly associated with age and independently corelated to all-cause (hazard ratio [HR] 1.31, *P* = 0.02) and cardiovascular mortality (HR 1.49, *P* = 0.04). A trend toward mortality risk in higher CMV IgG titer individuals was also observed. Importantly, PC3 retained prognostic value independent of chronological age, suggesting that immune dysfunction may contribute to excess mortality in dialysis patients.

**Conclusions:**

Our results confirmed that an age-associated immune signature was associated with all-cause and cardiovascular mortality in hemodialysis patients. This immune monitoring may be extended to other chronic disease populations associated with aging.

**Supplementary Information:**

The online version contains supplementary material available at 10.1186/s12979-025-00554-4.

## Introduction

Immune dysfunction is a hallmark of end-stage kidney disease[1], in which chronic low-grade inflammation contributes to organ damage [2–4]. This chronic inflammation resembles age-related immune alterations[5], known as *inflammaging* [6]. Inflammaging is implicated in the pathogenesis of cardiovascular diseases [7] and cancers [8]. Mechanistically, aging of the immune system, or “immunosenescence,”[9] is characterized by telomere shortening[10], relative depletion of naïve T lymphocytes and accumulation of late-stage or possibly terminally differentiated T lymphocytes especially in the CD8 subset [11, 12] The alteration in immune cell composition, which may represent an “immune risk phenotype”[13] associated with adverse outcomes in the elderly is similarly observed in ESKD [14–16]. Thus, immune dysfunction, a shared characteristic of ESKD and aging[17, 18], may be a link between immunosenescence and mortality risk. Multiple immune cells are implicated in aging; both monocytes [19, 20] (innate immunity) and T lymphocytes [21] (adaptive immunity) sustain chronic inflammation in kidney disease[22], concomitantly with detrimental effects from accumulation of uremic toxins and dialysis procedures [1]. Given the markedly reduced life expectancy in ESKD patients[23, 24], immune dysfunction may be a crucial yet underrecognized determinant of survival.

Previous research on the associations between immunity and outcomes focused on single cell subsets: some studies have demonstrated that CD14 + CD16 + monocytes are associated with cardiovascular events [25] and mortality [26]; reductions in CD4 and CD8 naïve T cell counts have been linked to cardiovascular and infectious events [27]; higher numbers of differentiated CD8 T cells have been correlated with all-cause mortality [28]. While these separate studies suggested the potential of individual immune cell types to predict patient survival, a single subset can only indirectly reflect a coordinated immunity alteration in patients. Since both monocytes and lymphocytes are exposed to uremic toxins in plasma, a broader analysis incorporating multiple subsets may delineate immune dysfunction and predict patient outcomes more precisely. This systemic approach has been explored in healthy adults, in whom multidimensional cellular phenotyping captures immune changes longitudinally and predicts longevity[29], but it has yet to be applied in dialysis patients or other chronic diseases.

Thus, we hypothesize that integrating lymphocyte and monocyte subsets into a composite index enhances mortality prediction. To test this hypothesis, we conducted immunophenotyping in the iESRD (Immunity in ESRD) cohort[15], the largest ever initiated to investigate the effects of immune dysfunction in dialysis patients. Based on the cross-sectional baseline information of the iESRD cohort, we have reported a comprehensive immune-aging phenotype of these patients at baseline and found that the duration of dialysis years was positively correlated with immunosenescence [15]. Now, we further test the associations between immune cell patterns and survival outcomes in a longitudinal follow-up. To the best of our knowledge, this is the first study to directly demonstrate that a multi-immune cell constellation predicts all-cause mortality and cardiovascular death in hemodialysis patients.

## Materials and methods

### Study participants

The iESRD cohort was described previously [15]. Four hundred nine Taiwanese adult participants were recruited from Far Eastern Memorial Hospital (FEMH) and National Taiwan University Hospital Yunlin Branch (NTUH-YL). As a cohort study, sample size was determined by the number of eligible patients. All participants provided written informed consent. The study was conducted in accordance with the ethical principles of the Declaration of Helsinki.

The inclusion criteria were at least 20 years old and receiving hemodialysis for at least three months. The exclusion criteria included an active infection or hospitalization within the three months prior to enrollment. Patients were followed from December 2014 to December 2017 at FEMH and from April 2016 to April 2019 at NTUH-YL. This study was approved by the Research Ethics Committee of both institutions (FEMH 103084-E and NTUYL 201511092 RINA).

### Data collection

Patient demographics (age and sex) and comorbidities (diabetes, hypertension, malignancy, congestive heart failure, coronary artery disease, peripheral artery disease, stroke, and solid organ transplantation) were ascertained at baseline from medical records. Blood samples were collected before the midweek hemodialysis session for hematology, biochemistry, and the isolation of peripheral blood mononuclear cells (PBMCs). Participants were followed for three years to assess survival outcomes. The primary outcome of interest was all-cause mortality. For an exploratory purpose, secondary outcomes include cardiovascular and infection-related deaths. Causes of mortality were determined through chart reviews.

### Immune cell subset quantification

Complete blood counts with differential counts were performed using an LH780 hematology analyzer (Beckman Coulter). For immune cell subset quantification, PBMCs were isolated using Ficoll-Paque (GE Healthcare) gradient centrifugation, then stained with fluorescence-conjugated antibodies: CD3-AF700 (UCHT1, BioLegend), CD4-PerCP/Cy5.5 (OKT4, BioLegend), CD8-APC/Cy7 (SK1, BioLegend), CD45RA-Alexa488 (HI100, BioLegend), CCR7-APC (G043H7, BioLegend), CD86-PE (IT2.2, eBioscience), CD14-FITC (M5E2, BioLegend), and CD16-APC (3G8, BioLegend). Flow cytometry was conducted using a MoFlo-XDP Sorter (Beckman Coulter). The gating strategy was described previously [15]. As depicted in Supplementary Fig. S1, we identified singlets by forward scatter area and height, followed by gating of lymphocytes and monocytes based on forward and side scatter characteristics. For CD3-positive T lymphocytes, CD4 and CD8 T cells were separated, followed by subgrouping into naïve (CD45RA + CCR7+), central memory (CD45RA-CCR7+), effector memory (CD45RA-CCR7-), and terminally differentiated cells (CD45RA + CCR7-) for both CD4 and CD8 cells. The CD86-positive monocytes were divided into classical monocytes (CD14 + + CD16-), intermediate monocytes (CD14 + + CD16+), and non-classical monocytes (CD14 + CD16+). PBMC isolation, antibody staining, and flow cytometry analysis were conducted at FEMH. Anti-CMV IgG titers were measured using the Elecsys^®^ immunoassay (Roche).

### Statistical analysis

Data were summarized using means and standard deviations for continuous variables, frequencies for dichotomous variables, and medians with interquartile ranges for non-normally distributed continuous variables. Cox regression was used to explore variables that predicted all-cause mortality, cardiovascular mortality, or infection-related mortality. Among them, all-cause mortality was the pre-specified primary outcome, whereas cardiovascular-related and infection-related mortality were secondary and exploratory endpoints. Because the cause-specific death outcomes were considered exploratory, we did not correct for significance values among analyzing different causes of death. In the multivariable model assessing the association between immune status and mortality, covariates were selected a priori based on established clinical knowledge. We adjusted for age, sex, recruiting institute, diabetes mellitus, hemoglobin, albumin, and C-reactive protein levels. No automated stepwise (forward or backward) variable selection procedures were applied. The set was forced into the final model to ensure appropriate and transparent confounding control. Because the purpose is to test whether there is any (novel) component of immune phenotype that remain prognostic, a full adjustment of parameters was applied for a more robust test, Principal component analysis (PCA) was conducted to identify immune cell subset patterns; resulting principal components were named in order of the magnitude of the eigenvalues (PC1, PC2, PC3, etc.). Linear regression was applied to delineate the relationship between PC3 and age. Statistical analyses were conducted by GraphPad Prism version 10.4.

## Results

### Baseline characteristics of hemodialysis patients

Among 409 patients (Table 1), the average age was 61.7 years (range 29.5–89.1 years) and the mean duration of dialysis was 6.23 years. Seventy-five patients died during the study, including 27 due to cardiovascular disease, 33 due to infection, 8 due to malignancy, and 7 from other causes. Eleven patients were lost to follow-up following transfer to other institutes. Seven patients were seronegative for anti-CMV IgG. However, excluding the seronegative subjects in further analyses did not alter the results, presumably due to the limited representation in the study population. There were 16 patients who received prior kidney transplantation, yet none of them received immunosuppressants due to the failed kidney graft. Compared with survivors at the end of the three-year follow-up, patients who died were older. They also had a higher prevalence of diabetes mellitus, lower hemoglobin, total cholesterol, and albumin levels, along with higher CRP levels, implying a chronic inflammatory state, even without clinical disease at baseline. These alterations were not significant among the seven seronegative patients, compared with seropositive counterparts. Moreover, deceased patients had a trend toward elevated anti-CMV IgG titers, suggesting a potential adverse impact of CMV, possibly mediated by T lymphocyte alterations. The elevated ages in CMV seropositive patients highlight the intertwined effect between age, CMV, and adverse outcome. Therefore, we further analyzed leukocyte subsets to assess their immune status.

**Table 1:** Patient characteristics at baseline

	All (*n* = 409)		Alive (*n* = 334)		Dead (*n* = 75)		*P* value	CMV seropositive (*n* = 402)		CMV seronegative (*n* = 7)		*P* value
Age (years)	61.7	(12.2)	59.9	(11.9)	69.3	(10.3)	**< 0.01**	62.0	(12.0)	44.1	(11.9)	**< 0.01**
Sex (male%)	50.6		51.2		48.0		0.70	50.8		42.9		0.72
Medical history												
Diabetes mellitus (%)	44.5		41.3		58.7		**< 0.01**	45.0		14.3		0.14
Hypertension (%)	76.3		74.9		82.7		0.18	76.9		42.9		0.06
Duration of Dialysis (years)	6.23	(5.09)	6.16	(5.06)	6.55	(5.25)	0.56	6.20	(5.02)	8.27	(8.58)	0.55
Laboratory data												
Hemoglobin (g/dl)	10.9	(1.4)	11.0	(1.2)	10.4	(1.8)	**< 0.01**	10.9	(1.38)	10.7	(1.09)	0.62
Platelet (x 10^3^/µl)	190.4	(65.9)	191.5	(62.4)	185.5	(80.1)	0.48	190.0	(65.1)	215.0	(109.0)	0.57
WBC (/µl)	6407	(1962)	6403	(1918)	6421	(2160)	0.95	6407	(1960)	6393	(2207)	0.99
Neutrophil (/µl)	4205	(1625)	4195	(1570)	4248	(1863)	0.80	4202	(1626)	4362	(1729)	0.82
Lymphocyte (/µl)	1486	(537)	1492	(514)	1457	(635)	0.61	1488	(538)	1359	(543)	0.56
Monocyte (/µl)	397	(178)	390	(176)	429	(183)	0.09	399	(179)	317	(99)	0.07
Total cholesterol (mg/dl)	152	(36.7)	154	(37.1)	143	(33.5)	**0.02**	152	(36.6)	152	(44.4)	1.00
Triglyceride (mg/dl)	147	(95.1)	147	(94.0)	146	(100.7)	0.95	147	(95.3)	124	(86.0)	0.50
Albumin (g/dl)	4.02	(0.38)	4.08	(0.32)	3.77	(0.52)	**< 0.01**	4.02	(0.39)	4.20	(0.26)	0.12
nPCR (g/kg/day)^#^	1.20	(0.44)	1.23	(0.32)	1.05	(0.76)	**< 0.01**	1.19	(0.44)	1.64	(0.48)	0.05
C-reactive protein (mg/dl)	0.290	(0.120–0.768)	0.271	(0.110–0.661)	0.533	(0.190–1.720)	**< 0.01**	0.30	(0.120–0.770)	0.190	(0.020–0.740)	0.30
CMV IgG (U/ml)	382.1	(175.3–801.7.3.7)	375.8	(167.4–739.0)	469.4	(240.3–1021.0)	< 0.01*	389.5	(181.0–812.8)	0.150	(0–0.150)	< 0.01*

^#^nPCR was only available among 305 out of 409 patients.*Log-transformed Welch’s t test was performed for CMV IgG. Continuous variables are shown as mean and standard deviation. Dichotomous variables are shown as percentage. CRP and anti-CMV IgG are shown as median with interquartile range. *P* values represent comparisons between surviving and deceased individuals and between CMV seropositive and seronegative patients. nPCR, normalized protein catabolic rate; CMV, cytomegalovirus. To convert hemoglobin, albumin, and CRP g/dl to g/l, times 10. To convert total cholesterol mg/dl to mmol/L, times 0.0259. To convert triglyceride mg/dl to mmol/L, times 0.0113

### Immune cell subset composition in hemodialysis patients

We characterized lymphocyte and monocyte populations as detailed in Methods (Table 2). Deceased patients had significantly lower percentages of both CD3-positive and CD3-negative lymphocytes, suggesting a decreased representation of T cells, B cells, and NK cells. However, the absolute numbers of these cells are not significantly different. In contrast, both percentages and absolute counts of CD4 and CD8 naïve T cells were lower in non-survivors, who had higher percentages and counts of CD4 terminally differentiated cells whereas only percentages but not counts of CD8 terminally differentiated cells were significantly higher in non-survivors. No significant differences were seen for myeloid cells, neither neutrophils nor any population of monocytes. These findings suggest that a more differentiated T cell population is linked to poorer survival in ESKD.

**Table 2:** Baseline immune cell subset percentages and numbers

	All (*n* = 409)		Alive (*n* = 334)		Dead (*n* = 75)		*P* value
Percentage							
Neutrophil	64.7	(8.7)	64.7	(8.1)	64.46	(11.0)	0.80
CD3	60.7	(14.1)	61.5	(13.4)	57.1	(16.5)	**0.02**
CD3-negative lymphocyte	39.4	(14.1)	38.6	(13.4)	42.9	(16.5)	**0.02**
CD4	58.6	(10.5)	58.6	(10.6)	58.59	(9.7)	0.98
Naïve T cells	31.1	(12.9)	32.1	(12.6)	26.84	(13.4)	**< 0.01**
Central memory T cells	43.8	(10.6)	43.9	(10.6)	43.09	(11.0)	0.54
Effector memory T cells	23.3	(11.3)	22.3	(10.7)	27.75	(12.9)	**< 0.01**
Terminally differentiated T cells	1.8	(2.1)	1.7	(1.8)	2.33	(3.1)	**0.02**
CD8	29.4	(9.9)	29.2	(9.9)	29.49	(9.9)	0.89
Naïve T cells	20.4	(14.5)	21.8	(14.7)	14.13	(11.7)	**< 0.01**
Central memory T cells	4.7	(4.4)	4.8	(4.5)	4.39	(3.7)	0.49
Effector memory T cells	38.1	(14.5)	37.5	(14.0)	40.30	(16.6)	0.14
Terminally differentiated T cells	36.8	(15.5)	35.9	(15.4)	41.18	(15.3)	**0.01**
Monocyte	6.3	(2.0)	6.2	(2.0)	6.788	(1.9)	**0.02**
Classical monocyte	65.8	(11.7)	65.6	(11.6)	66.43	(12.3)	0.60
Intermediate monocyte	9.8	(6.2)	9.7	(6.0)	10.16	(7.1)	0.53
Non-classical monocyte	14.6	(8.8)	14.8	(8.7)	13.7	(9.2)	0.34
Absolute number							
Neutrophil	4205	(1625)	4195	(1570)	4248	(1863)	0.80
CD3	907.4	(414.1)	918.4	(387.8)	858.6	(515.3)	0.26
CD3-negative lymphocyte	578.2	(288.1)	573.6	(287.1)	598.7	(293.7)	0.50
CD4	523	(232.6)	529.8	(218.5)	492.7	(287.2)	0.21
Naïve T cells	164.5	(111.9)	171.6	(110.2)	133.1	(114.3)	**0.01**
Central memory T cells	228.6	(116.2)	233.1	(113.1)	208.4	(127.8)	0.10
Effector memory T cells	120.6	(86.4)	116.4	(74.1)	139.6	(126.6)	**0.04**
Terminally differentiated T cells	9.3	(11.7)	8.7	(9.5)	11.7	(18.6)	**0.04**
CD8	275	(180.2)	277.6	(177.1)	263.6	(194.2)	0.54
Naïve T cells	54.5	(61.9)	59.4	(65.5)	32.7	(35.5)	**< 0.01**
Central memory T cells	12.1	(13.9)	12.6	(14.4)	9.9	(11.5)	0.13
Effector memory T cells	102.5	(83.6)	101.2	(76.7)	108.3	(109.5)	0.51
Terminally differentiated T cells	105.8	(95.2)	104.3	(95.0)	112.7	(96.8)	0.49
Monocyte	397.4	(177.9)	390.3	(176.3)	429.1	(182.5)	0.09
Classical monocyte	264.4	(141.6)	259.1	(141.3)	287.9	(141.7)	0.11
Intermediate monocyte	40.4	(33.9)	39.2	(31.7)	45.7	(42.1)	0.13
Non-classical monocyte	56.3	(38.2)	56.1	(37.8)	57.4	(40.3)	0.78

Absolute numbers were expressed in count per µl. Immune cell subset numbers and percentages were shown as mean and standard deviation. *P* values represent unpaired *t*-tests between surviving and deceased individuals

### Aging-associated immune cell alterations in hemodialysis patients

To explore the nature of immune cell subsets, we investigated whether immune cell numbers correlate with age (Table 3). Increased age was associated with lower CD4 and CD8 T lymphocyte counts, although trends in their corresponding percentages appeared less distinct. Higher monocyte percentages and counts were also correlated with aging. Among T cell subsets, increased age correlated with fewer naïve and more effector memory and terminally differentiated cells, in both the CD4 and CD8 T cells, whether as percentages or absolute numbers. All three monocyte subsets also showed age-related accumulation in absolute numbers, paralleling the trend of total monocyte counts, while monocyte subset distribution differences were less prominent in terms of percentage. These findings support the association between immune cell alterations and aging. The overlap between the mortality-associated (Table 2) and age-associated immune cell subsets (Table 3) suggests that immunosenescence may serve as a link between aging and mortality.

**Table 3:** Association between immune cell subset numbers and age

	*ρ*	95% CI	*P* value
Percentage			
Neutrophil	0.02	−0.08 to 0.12	0.70
CD3-negative lymphocyte	0.14	0.04 to 0.24	**< 0.01**
CD4	−0.04	−0.14 to 0.06	0.40
Naïve T cells	−0.24	−0.33 to −0.15	**< 0.01**
Central memory T cells	−0.04	−0.14 to 0.06	0.38
Effector memory T cells	0.24	0.15 to 0.34	**< 0.01**
Terminally differentiated T cells	0.17	0.08 to 0.27	**< 0.01**
CD8	0.00	−0.10 to 0.10	0.97
Naïve T cells	−0.51	−0.58 to −0.44	**< 0.01**
Central memory T cells	−0.02	−0.12 to 0.08	0.67
Effector memory T cells	0.14	0.04 to 0.24	**< 0.01**
Terminally differentiated T cells	0.32	0.23 to 0.41	**< 0.01**
Monocyte	0.18	0.08 to 0.28	**< 0.01**
Classical monocyte	−0.02	−0.12 to 0.08	0.68
Intermediate monocyte	0.07	−0.03 to 0.17	0.16
Non-classical monocyte	0.01	−0.09 to 0.11	0.88
Absolute number			
Neutrophil	0.05	−0.05 to 0.14	0.36
CD3-negative lymphocyte	0.11	0.01 to 0.21	**0.03**
CD4	−0.11	−0.21 to −0.01	**0.02**
Naïve T cells	−0.26	−0.35 to −0.16	**< 0.01**
Central memory T cells	−0.11	−0.20 to −0.01	**0.03**
Effector memory T cells	0.12	0.02 to 0.22	**0.01**
Terminally differentiated T cells	0.12	0.02 to 0.21	**0.02**
CD8	−0.07	−0.17 to 0.03	0.14
Naïve T cells	−0.49	−0.56 to −0.41	**< 0.01**
Central memory T cells	−0.09	−0.19 to 0.01	0.08
Effector memory T cells	0.01	−0.09 to 0.11	0.89
Terminally differentiated T cells	0.14	0.04 to 0.24	**< 0.01**
Monocyte	0.17	0.07 to 0.26	**< 0.01**
Classical monocyte	0.13	0.03 to 0.23	**0.01**
Intermediate monocyte	0.14	0.04 to 0.23	**0.01**
Non-classical monocyte	0.11	0.01 to 0.21	**0.02**

Correlation between immune cell subsets and age. *ρ*, Spearman correlation coefficient. 95% *CI,* 95% confidence interval

### Associations between individual immune cell subset counts and mortality

We then examined whether immune cell percentages and counts independently predicted mortality (Supplementary Table S1). In univariable Cox regression, CD4 and CD8 naïve cell percentages and counts were negatively associated with mortality, while CD4 effector memory and terminally differentiated cell percentages and absolute counts were positively associated with mortality. In addition, CD3-negative lymphocyte and CD8 terminally differentiated cell percentages were also mortality risk factors.

For multivariable regression in Model 1, after adjusting for age, sex, and institute, CD4 naïve cell percentage, CD4 effector memory cell percentage, and CD8 naïve cell count remained statistically significant. However, in Model 2, which additionally adjusted for hemoglobin, diabetes, albumin, and CRP, only the number of CD4 effector memory cells emerged as a significant predictor of mortality. The inconsistency among different models may indicate the instability of models with borderline effect sizes for individual immune cell subsets.

We move further to secondary outcomes of either cardiovascular or infection-related deaths thereafter. Similar inconsistency was noted when considering cardiovascular mortality. In univariable Cox regression, both percentages and absolute counts of CD4 effector memory cells predicted events. In contrast, CD8 naïve, CD8 terminally differentiated cells, and intermediate monocytes were significant predictors only in terms of percentage, while absolute counts of CD4 terminally differentiated cells and non-classical monocytes were also significant predictors. For model 2, non-classical monocyte percentages and counts were both cardiovascular mortality risk factors, along with CD4 effector memory cell counts.

For infection-related mortality, decreased CD4 and CD8 naïve cells were significant predictors in univariable regression, for either their percentages or counts. CD4 effector memory and terminally differentiated cell percentage were also predictors. With adjustment in model 1, CD4 naïve cell and non-classical monocyte percentages and counts were negatively correlated with infection-related death. However, only CD4 naïve cell counts remained a consistent protective predictor in Model 2.

Among the cell subsets associated with mortality (Table 2) and those correlated with age (Table 3), CD4 effector memory cell counts remained all-cause and cardiovascular mortality predictors independent of age. Non-classical monocyte percentages and counts were cardiovascular death but not all-cause mortality predictors, but CD4 naïve cell counts acted as a protector for infection-related death independent of age. These findings highlight a complex interplay between age-dependent and age-independent effects of immune cells. Meanwhile, repeated analysis to multiple subsets may confer risk to false positives. Individually, immune cell subsets provide limited predictive value for mortality.

### Principal component analysis of immune cell subset patterns

**Fig. 1:**
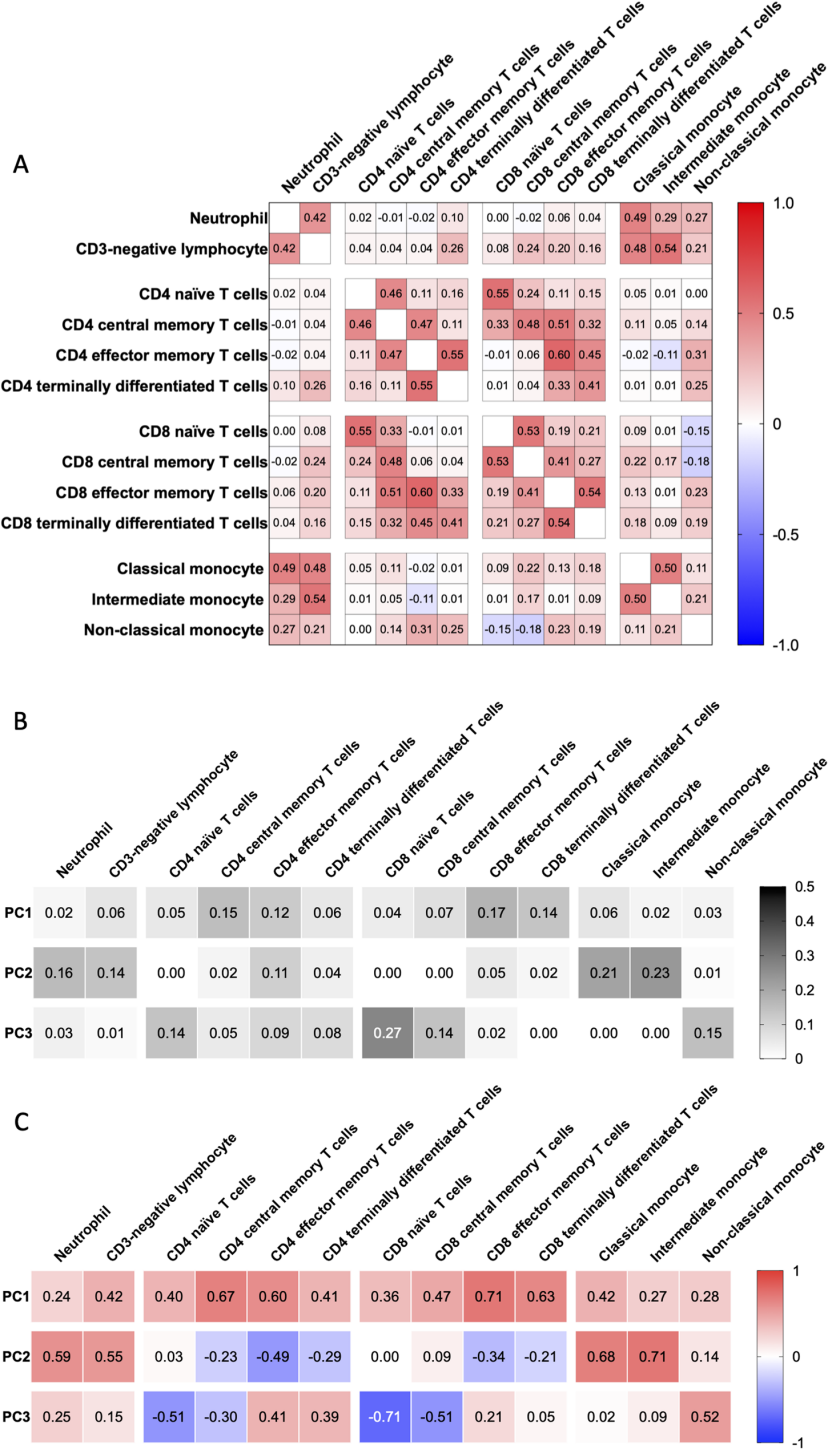
Principal Component Analysis of Immune Cell Subset Numbers. **A** Correlogram displaying pairwise comparisons among 13 immune cell subsets. The number in each cell represents Spearman correlation coefficient, with colors ranging from red (+ 1.0) to blue (−1.0) based on correlation coefficient. **B** Contribution of each immune cell subset to the Principal Components. The number in each cell represents the proportion of contribution. The grayscale reflects the magnitude of their contribution. **C** Correlation between immune cell subset numbers to the Principal Components. The number in each cell represents Spearman correlation coefficient, with colors ranging from red (+ 1.0) to blue (−1.0) based on correlation coefficient

We further hypothesized that summarizing immune cell counts into patterns of cell distribution enhances their robustness of mortality prediction. First, we examined the correlation matrix of immune cell counts (Fig. 1a). Several subsets showed positive correlations, such as CD4 and CD8 naïve cells, as well as CD4 and CD8 effector memory cells. Conversely, non-classical monocyte counts negatively correlated with CD8 naïve and CD8 central memory cell numbers.

Next, we applied PCA to define immune cell subset patterns. Three principal components were extracted, namely PC1, PC2, and PC3, which explained 22.83%, 16.78%, and 14.25% of the variance, respectively. The contributing proportion (Fig. 1b) and correlation coefficient (Fig. 1c) of each immune cell subset to the principal component were reported. Specifically, a higher PC1 corresponded to higher lymphocyte counts (ρ = 0.92) and was equally contributed by CD4 central memory, CD4 effector memory, CD8 effector memory, and CD8 terminally differentiated cell numbers. A higher PC2 was linked to increased neutrophils, CD3-negative lymphocytes, classical monocytes, and intermediate monocytes, whereas CD4 and CD8 effector memory T cells were reduced. Meanwhile, PC2 also showed correlation with total leukocyte and monocyte counts (ρ = 0.60 and 0.66, respectively). Finally, a higher PC3 was associated with decreased CD4 naïve, CD4 central memory, CD8 naïve, and CD8 central memory cells, along with increased CD4 effector memory, CD8 effector memory cells, and non-classical monocytes. Notably, PC3 emerged independently of extrinsic variables yet strongly aligned with age-associated immune cell changes (Table 3). Overall, the thirteen immune cell subsets were condensed into three principal components, capturing 53.85% of the variance.

### Principal component scores and baseline characteristics

We then explored correlations between principal component scores and baseline clinical parameters (Supplementary Table S2). PC1 showed a positive correlation with hemoglobin, leukocyte count, serum albumin, and diabetes diagnosis. PC2 was positively correlated with hemoglobin, leukocyte count, CRP, and diabetes diagnosis. Interestingly, PC3 was positively correlated with age (ρ = 0.35, *P* < 0.01), leukocyte count, CRP, and log-transformed anti-CMV IgG titers, while it was negatively correlated with hemoglobin and albumin. We also assessed the association between log-transformed anti-CMV IgG titers and other parameters. Log-transformed CMV IgG titer correlated with age (ρ = 0.12, *P* = 0.02) and low hemoglobin. These findings suggest that PC3 and anti-CMV IgG titers may both function as markers of aging.

### Associations between immune cell subset patterns and survival outcomes

**Fig. 2:**
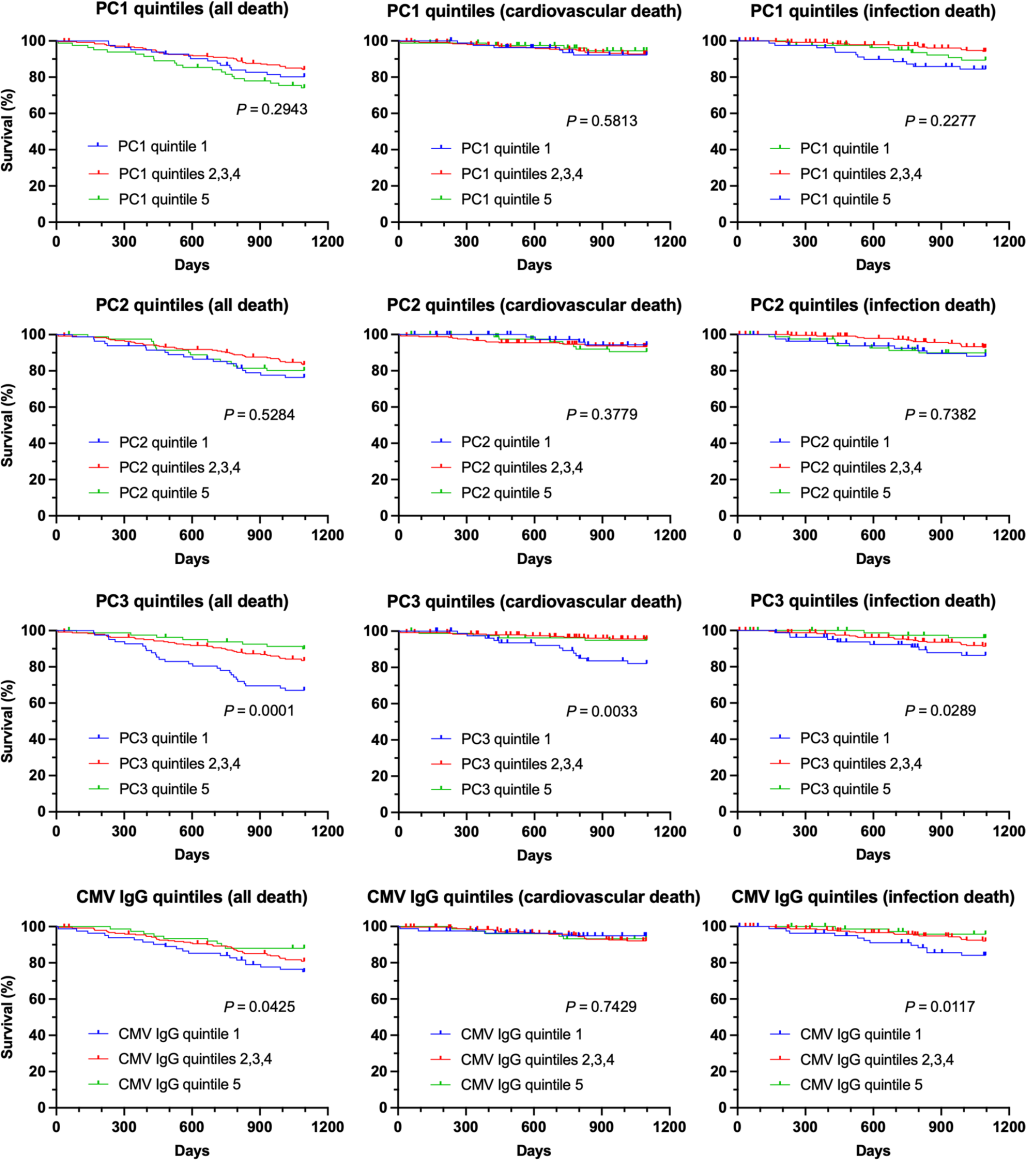
Patient survival based on principal component quintiles and CMV IgG quintiles. Patients were grouped according to quintiles of Principal Component (PC1, PC2, and PC3) scores and anti-CMV IgG titers. Kaplan-Meier survival curves were generated for all-cause mortality, cardiovascular death, and infection-related death. P values represent log-rank tests for trend, demonstrating differences among survival curves. Higher PC3 and CMV IgG quintiles were associated with poorer patient survival. CMV IgG seronegative subjects were excluded from the CMV IgG quintile analysis

To further investigate whether immune cell profiles are associated with clinical outcomes, patients were stratified into three groups (Fig. 2): (1) the highest quintile of principal component scores, (2) the second, third, and fourth quintiles combined, and (3) the lowest quintile. For PC1-based and PC2-based groupings, survival curves for all-cause mortality, cardiovascular mortality, and infection-related mortality did not differ significantly between groups. In contrast, patients in the highest PC3 quintile had the poorest overall survival (*P* < 0.01). Additional exploratory analysis demonstrated that the highest PC3 quintile also possessed highest cardiovascular mortality (*P* < 0.01) and highest infection-related mortality (*P* = 0.03), compared to other PC3 quintiles. Similar to Supplementary Table S2, when examining the baseline characteristics among PC3 quintile groups (Supplementary Table S3), patients with highest PC3 scores were associated older age, more anemic, more neutrophils, lower albumin, higher CRP, and higher CMV IgG titer. This highlighted the features of PC3 as aging and inflammation markers.

At baseline, patients who died during follow-up tended to have higher CMV IgG titers (Table 1). Consequently, we also stratified patients into quintiles based on the CMV IgG titer. CMV IgG titer was a significant predictor of overall survival and infection-related mortality (*P* = 0.04 and 0.01, respectively), but not cardiovascular death. These results may indicate that immune status, as captured by PC3 or CMV IgG titer—both aging-associated parameters— was linked to mortality of different pathogenesis.

However, the principal components and CMV IgG titers were correlated with baseline characteristics (Supplementary Table S2). To assess independent effects, we performed multivariable Cox regression (Table 4). In Model 1, age, sex, and institute were adjusted. Model 2 was composed of hemoglobin, diabetes diagnosis, albumin, and CRP in addition to Model 1 variables. Model 3 included the log CMV IgG titer in addition to Model 2 variables. For all-cause mortality, PC3 was a significant predictor in univariable, multivariable Model 1, 2, and 3. For cardiovascular mortality, PC2 was not significant in univariable or Model 1 but reached significance in Model 2 and Model 3. In contrast, PC3 consistently predicted cardiovascular death in univariable, Model 1, and Model 3 (with marginal significance in Model 2). Regarding infection-related mortality, both PC3 and CMV IgG titers were significant in univariable regression but not in multivariable models. These findings establish immune status, captured by PC3, as an independent risk factor of mortality in hemodialysis patients.

**Table 4:** Association between principal components and CMV IgG titers with all-cause mortality, cardiovascular death, and infection-related death

	Univariable			Model 1				Model 2				Model 3*		
	HR	95% CI	*P* value	HR	95% CI	*P* value		HR	95% CI	*P* value		HR	95% CI	*P* value
All-cause death														
Principal Component 1	1.00	0.87 to 1.14	0.95	0.99	0.86 to 1.12	0.84		1.02	0.89 to 1.16	0.79		1.01	0.88 to 1.15	0.87
Principal Component 2	1.04	0.89 to 1.21	0.62	0.99	0.83 to 1.17	0.87		0.89	0.74 to 1.06	0.20		0.89	0.75 to 1.06	0.21
Principal Component 3	1.49	1.23 to 1.79	**< 0.01**	1.43	1.15 to 1.78	**< 0.01**		1.33	1.06 to 1.66	**0.01**		1.31	1.05 to 1.66	**0.02**
Log CMV IgG*	1.55	0.97 to 2.51	0.07	1.29	0.80 to 2.11	0.30		1.24	0.76 to 2.05	0.39				
Cardiovascular death														
Principal Component 1	1.19	0.97 to 1.43	0.08	1.18	0.96 to 1.42	0.09		1.17	0.95 to 1.40	0.11		1.17	0.96 to 1.41	0.10
Principal Component 2	0.82	0.62 to 1.08	0.17	0.82	0.61 to 1.10	0.18		0.73	0.55 to 0.99	**0.04**		0.73	0.54 to 0.98	**0.04**
Principal Component 3	1.61	1.18 to 2.19	**< 0.01**	1.51	1.05 to 2.20	**0.03**		1.45	1.01 to 2.11	0.05		1.50	1.03 to 2.21	**0.04**
Log CMV IgG*	1.06	0.50 to 2.31	0.88	0.88	0.40 to 1.97	0.75		0.91	0.42 to 2.02	0.82				
Infection death														
Principal Component 1	0.90	0.72 to 1.11	0.35	0.88	0.70 to 1.08	0.26		0.93	0.74 to 1.14	0.51		0.89	0.70 to 1.11	0.33
Principal Component 2	0.98	0.76 to 1.24	0.87	0.90	0.68 to 1.17	0.46		0.86	0.65 to 1.12	0.28		0.86	0.65 to 1.13	0.28
Principal Component 3	1.51	1.14 to 2.01	**< 0.01**	1.38	1.01 to 1.92	0.05		1.31	0.95 to 1.82	0.10		1.23	0.90 to 1.73	0.21
Log CMV IgG*	2.55	1.23 to 5.39	**0.01**	2.05	0.97 to 4.38	0.06		1.99	0.90 to 4.44	0.09				

*CMV seronegative subjects excluded. Univariable and multivariable Cox regression

Model 1 was adjusted for age, sex, and institute

Model 2 was adjusted for age, sex, institute, hemoglobin, diabetes mellitus, albumin, and C-reactive protein

Model 3 included all parameters from Model 2, with an addition of log-transformed CMV IgG titers

For the check of multicollinearity among variables, VIFs (variance inflation factor) were all < 5 in model 1. For model 2 or model 3, those with higher VIF include age, hemoglobin, albumin, and CMV IgG. Consequently, model 4 was constructed, retaining age in addition to other low VIF variables. In that, all VIFs were below 5, without affecting the significance of PC3 on outcome prediction, confirming robustness of the finding (Supplementary Table S4).

### Comparison between immune signature model to single cell subset on association on mortality outcome

To appreciate the improvement in outcome prediction provided by the composite immune signature compared with individual cell subsets or clinical variables alone, we performed AICc-based model comparison between either immune signature or single cell subset (naive CD4 T cell or naive CD8 T cell) as the parameter of interest, utilizing multicollinearity-free model 4. The PC3 immune-signature model was the top-ranked model (ΔAICc = 0.00, Akaike weight w_i_ = 0.55) and received 55% of the total model support (Supplementary Table S5). It outperformed the next-best single cell model (naive CD8-only) by an evidence ratio of 1.3 and the clinical baseline model by an evidence ratio of 11.8.

### Characterization of immune signature among different causes of death

The prognostic significance of principal components on survival was depicted in Fig. 2 and Table 4. Furthermore, we were also interested in whether patients who died of different causes possessed distinct immune signatures. Thus, distribution of principal component scores was compared between deaths caused by cardiovascular diseases, infectious diseases, and oncologic diseases (Supplementary Fig. S2). However, statistical tests between groups did not show significant differences, either among the three groups or between pair-wise comparisons. This may indicate the effect of immune profile per se may need to be considered in concert with other parameters.

### Partitioning the age-independent effect of PC3 on mortality

PC3 was identified as an independent risk factor of mortality (Table 4). However, PC3 was positively correlated with age (Supplementary Table 1), suggesting that its association with mortality might be partially explained by age-related immune changes. Whereas immune ageing was partly driven by chronological age, the persistent association of the PC3 with mortality after adjustment for age and other variables suggested that additional age-independent pathways contributed to the immune phenotype and its prognostic impact. To better understand additional age-independent effects, we examined the distribution of PC3 across different ages (Supplementary Fig. S3A). Using simple linear regression, we estimated the expected PC3 value based on age (PC3 = 0.03615 × age − 2.229, R² = 0.1043). The difference between the actual PC3 and the age-expected PC3 (‘PC3 residual’) represents the age-independent immune component.

To assess the prognostic value of the age-independent PC3 component, we substituted PC3 with the PC3 residual in Cox regression models. The PC3 residual demonstrated the same hazard ratios and statistical significance as PC3 in both Model 1 and Model 2, both including age as an independent variable. This confirms that the association between PC3 and mortality is not solely driven by age but also reflects intrinsic immune dysfunction.

To further illustrate this effect, we estimated an immune-adjusted age, a hypothetical age accounting for the combined risk of death predicted from age and PC3 residual. To assess the contribution of PC3 to estimated risk of death, we generated a scatter plot to demonstrate how this immune-adjusted age deviates from the chronological age (Supplementary Fig. S3B). Wherever an immune-adjusted age was higher than their chronological age, it suggested an accelerated immunosenescence outpaced the age-predicted extent of immune aging, with an equivalent age difference of 0.36 (IQR − 3.52 to + 3.50) years. These findings indicate that immune status, as reflected by PC3, varies among patients independent of their age and may serve as a mortality risk determinant in hemodialysis patients.

Overall, our findings highlight the clinical relevance of immune profiling in hemodialysis patients and suggest that immune dysfunction, as captured by PC3, enhances risk stratification beyond chronological age.

## Discussion

The immune system is critical for health and has long been recognized for its impact on disease outcomes. However, the clinical assessment of immune function has largely been confined to identifying states of immunodeficiency. This limited scope fails to account for the nuanced yet clinically significant immune alterations occurring in chronic diseases, which may contribute to impaired outcomes. Our findings indicate that a specific pattern of immune cell composition independently predicts all-cause mortality and cardiovascular death in ESKD. This principal component reflects both aging and additional age-independent immune dysfunction, underscoring the role of immune alterations in patient survival. As a novel example of immune profiling for outcome prediction in ESKD, our study demonstrates a pattern-based approach to provide a comprehensive view of immune dysfunction and its independent contribution to mortality.

While immune cell alterations in ESKD have been documented[14, 30], most analyses have focused on a limited number of cell types [25, 26, 28, 31–33]. However, immune cell subsets can exhibit strong correlations (Fig. 1a); thus, associations based on a single subset may be confounded by interrelated immune dynamics. This highlights the need for multidimensional profiling to capture the coordinated alterations in the immune system. For example, while CD4 effector memory cells were associated with mortality (Supplementary Table S1), they were not the strongest contributor to death-predicting PC3 (Fig. 1c). This suggests that although CD4 effector memory cell expansion reflects one aspect of immune status, the broader immune landscape, including other cells, better predicts adverse outcomes.

Although we did not incorporate heathy subjects as a reference for comparison, the dialysis patients-derived PC3 still presented a striking similarity to known immune alteration observed in dialysis patients and aging individuals. Xiang et al. reported that decreased naive T cells, decreased CD4 naive T cells, and accumulation of CD8 memory T cells were associated with mortality in dialysis patients [28]. Research from the same group also pointed out that decreased CD4 naive T cells were associated with cardiovascular events, whereas decreased CD8 naive T cells were associated with infection events [27]. The similar yet non-identical prognostic values of various naive and differentiated T cells reflected the complex interplay between immune cell subsets, supporting the utilization of summative tool to capture the whole picture at the same time. In line with this, a report from Heine et al. showed that monocyte 2 was associated with cardiovascular events [25]. Along with the known premature aging of immune system in dialysis patients [15, 16] and the reported immune aging effect on prognosis in heathy individuals, [29] we believed our result added support for immune aging acting as the cause of premature death in dialysis patients,

Besides death-predicting PC3, PC1 correlated with total lymphocyte count, a susceptibility marker for virus infections [34] and malignancies [35]. Consistently, a higher PC1 score showed a trend toward protection against infection-related mortality (Table 4), likely reflecting the role of lymphocytes to combat infection, whereas PC2 was associated with total leukocyte, neutrophil and monocyte counts, likely reflecting innate immune status. In survival analyses (Fig. 2), mid-PC1 quintiles and mid-PC2 quintiles were better indicators than the highest or lowest quintiles. This may represent the advantage of maintaining homeostasis, which cannot be captured by proportional hazard models.

The overrepresentation of non-classical monocytes in PC3 aligns with their role in inflammaging[36], atherosclerotic cardiovascular disease[37, 38], and frailty with premature death [6]. In coronary artery disease, non-classical monocytes have been associated with endothelial dysfunction[39], which may also be driven by senescent T lymphocytes[40], particularly in a uremic environment[41], illustrating a concerted effect of monocytes and T lymphocytes on immunosenescence-related pathology.

The distinctions between PC3 and CMV IgG titers are intriguing. Increased age was associated with both parameters being higher (Supplementary Table 1). The highest quintiles of PC3 and CMV IgG titer were both associated with the poorest survival (Fig. 2). Nevertheless, after adjustment, PC3 independently predicted all-cause and cardiovascular mortality, whereas CMV IgG titers were associated with infection-related death only in univariable analysis. While both reflect immunosenescence, possibly driven by CMV infection, PC3 may capture a broader immune cell landscape, whereas CMV IgG titer primarily reflects humoral response, a restricted component of immunity.

While iESRD represents the largest cohort dedicated to investigating immunity in hemodialysis patients, our study has several limitations. The event numbers of cause-specific mortality were limited; their corresponding analyses should be interpreted with caution. We did not incorporate external cohorts to validate the predictive ability of immune cell patterns. Additionally, given the CMV seropositivity of our subjects, cell patterns may differ in seronegative patients, of which had too few (only seven) for analysis. Meanwhile, BMI was not included in the data set, which may also reflect the protein-energy wasting status, additionally contributing to chronic inflammation. Other immune cells not definitively identified, such as B cells, NK cells, and myeloid-derived suppressor cells, may represent additional aspects of immune dysfunction [31, 42, 43]. Finally, generalization into other ethnicities should consider the nature of our Taiwanese cohort.

In conclusion, we identified distinct aging-associated immune cell patterns in ESKD, characterized by the greater loss of naïve cells and increased accumulation of differentiated T lymphocytes and non-classical monocytes. This immune profile was linked to mortality independently of other risk factors. Our findings highlight the coordinated nature of immune alterations in dialysis patients and underscore the potential clinical utility of immune profiling. Future research is needed to determine whether immune monitoring can be successfully applied in other chronic disease populations.

## Supplementary Information

Supplementary Material 1.

## Data Availability

Raw data are not publicly available due to the confidentiality statement in the consent form approved by the ethical committee of Far Eastern Memorial Hospital. Anonymized participant data, flow cytometry files, and statistical analysis results are available upon reasonable request to the corresponding author after approval by the ethical committee of Far Eastern Memorial Hospital.
