# Supplementary material for: Aging-associated immune signature as a predictor of mortality in end-stage renal disease: results from the longitudinal iESRD study
Source: Immun Ageing. 2025 Dec 29;23:4. doi: 10.1186/s12979-025-00554-4 (PMC12860072; doi:10.1186/s12979-025-00554-4)

| **Supplementary Table S1.** Associations between immune cell subsets and all-cause mortality, cardiovascular death, or infection death | | | | | | | | | | | | | | | | | | | |
| --- | --- | --- | --- | --- | --- | --- | --- | --- | --- | --- | --- | --- | --- | --- | --- | --- | --- | --- | --- |
|  | **Percentage** | | | | | | | | |  | **Absolute number** | | | | | | | | |
|  | Univariable | | | Model 1 | | | Model 2 | | |  | Univariable | | | Model 1 | | | Model 2 | | |
|  | HR | 95% CI | P value | HR | 95% CI | P value | HR | 95% CI | P value |  | HR | 95% CI | P value | HR | 95% CI | P value | HR | 95% CI | P value |
| **All-cause death** |  |  |  |  |  |  |  |  |  |  |  |  |  |  |  |  |  |  |  |
| Neutrophils | 1.00 | 0.97 to 1.02 | 0.84 | 1.00 | 0.97 to 1.02 | 0.76 | 0.98 | 0.95 to 1.00 | 0.10 |  | 1.00 | 1.00 to 1.00 | 0.78 | 1.00 | 1.00 to 1.00 | 0.94 | 1.00 | 1.00 to 1.00 | 0.24 |
| CD3-negative lymphocytes | 1.02 | 1.00 to 1.04 | **0.01** | 1.00 | 0.98 to 1.03 | 0.76 | 1.00 | 0.98 to 1.02 | 0.84 |  | 1.00 | 0.99 to 1.01 | 0.52 | 1.00 | 0.99 to 1.01 | 0.89 | 1.00 | 0.99 to 1.01 | 0.77 |
| CD4 |  |  |  |  |  |  |  |  |  |  |  |  |  |  |  |  |  |  |  |
| Naïve T cells | 0.97 | 0.95 to 0.99 | **0.00** | 0.98 | 0.96 to 1.00 | **0.03** | 0.98 | 0.97 to 1.00 | 0.10 |  | 0.96 | 0.94 to 0.99 | **0.01** | 0.98 | 0.95 to 1.00 | 0.08 | 0.99 | 0.96 to 1.01 | 0.31 |
| Central memory T cells | 0.99 | 0.97 to 1.01 | 0.54 | 1.00 | 0.98 to 1.02 | 0.84 | 1.00 | 0.98 to 1.02 | 0.89 |  | 0.98 | 0.96 to 1.00 | 0.09 | 0.99 | 0.97 to 1.01 | 0.36 | 1.00 | 0.98 to 1.02 | 0.88 |
| Effector memory T cells | 1.03 | 1.02 to 1.05 | **0.00** | 1.02 | 1.00 to 1.04 | **0.02** | 1.02 | 1.00 to 1.04 | 0.09 |  | 1.03 | 1.00 to 1.05 | **0.03** | 1.02 | 0.99 to 1.04 | 0.12 | 1.03 | 1.00 to 1.05 | **0.02** |
| Terminally differentiated T cells | 1.11 | 1.02 to 1.22 | **0.01** | 1.08 | 0.99 to 1.17 | 0.10 | 1.05 | 0.97 to 1.15 | 0.25 |  | 1.17 | 0.99 to 1.32 | **0.03** | 1.13 | 0.96 to 1.27 | 0.09 | 1.13 | 0.97 to 1.27 | 0.08 |
| CD8 |  |  |  |  |  |  |  |  |  |  |  |  |  |  |  |  |  |  |  |
| Naïve T cells | 0.96 | 0.94 to 0.98 | **0.00** | 0.97 | 0.95 to 1.00 | 0.05 | 0.99 | 0.96 to 1.01 | 0.23 |  | 0.86 | 0.79 to 0.93 | **<0.01** | 0.91 | 0.83 to 0.98 | **0.03** | 0.95 | 0.88 to 1.00 | 0.11 |
| Central memory T cells | 0.98 | 0.92 to 1.04 | 0.52 | 0.97 | 0.90 to 1.04 | 0.37 | 0.95 | 0.88 to 1.03 | 0.21 |  | 0.85 | 0.65 to 1.03 | 0.14 | 0.82 | 0.63 to 1.01 | 0.10 | 0.82 | 0.61 to 1.03 | 0.13 |
| Effector memory T cells | 1.01 | 1.00 to 1.03 | 0.14 | 1.01 | 0.99 to 1.03 | 0.21 | 1.01 | 0.99 to 1.02 | 0.44 |  | 1.01 | 0.98 to 1.03 | 0.56 | 1.01 | 0.98 to 1.03 | 0.71 | 1.01 | 0.98 to 1.03 | 0.51 |
| Terminally differentiated T cells | 1.02 | 1.01 to 1.03 | **0.01** | 1.00 | 0.99 to 1.02 | 0.62 | 1.00 | 0.99 to 1.02 | 0.58 |  | 1.01 | 0.98 to 1.03 | 0.51 | 1.00 | 0.97 to 1.02 | 0.73 | 1.00 | 0.98 to 1.02 | 0.95 |
| Monocyte |  |  |  |  |  |  |  |  |  |  |  |  |  |  |  |  |  |  |  |
| Classical monocytes | 1.01 | 0.99 to 1.03 | 0.60 | 1.00 | 0.98 to 1.03 | 0.76 | 1.00 | 0.98 to 1.02 | 0.98 |  | 1.01 | 0.99 to 1.02 | 0.11 | 1.01 | 0.99 to 1.02 | 0.45 | 1.00 | 0.98 to 1.01 | 0.94 |
| Intermediate monocytes | 1.01 | 0.98 to 1.05 | 0.46 | 1.00 | 0.96 to 1.04 | 0.89 | 1.00 | 0.96 to 1.04 | 0.99 |  | 1.05 | 0.98 to 1.11 | 0.11 | 1.02 | 0.95 to 1.09 | 0.48 | 1.01 | 0.94 to 1.08 | 0.79 |
| Non-classical monocytes | 0.99 | 0.96 to 1.01 | 0.32 | 0.99 | 0.95 to 1.02 | 0.46 | 1.00 | 0.96 to 1.03 | 0.86 |  | 1.01 | 0.95 to 1.07 | 0.80 | 1.01 | 0.94 to 1.09 | 0.70 | 1.01 | 0.93 to 1.08 | 0.86 |
|  |  |  |  |  |  |  |  |  |  |  |  |  |  |  |  |  |  |  |  |
| **Cardiovascular death** |  |  |  |  |  |  |  |  |  |  |  |  |  |  |  |  |  |  |  |
| Neutrophils | 0.97 | 0.93 to 1.01 | 0.10 | 0.96 | 0.92 to 1.00 | 0.08 | 0.95 | 0.91 to 0.99 | **0.02** |  | 1.00 | 1.00 to 1.00 | 0.91 | 1.00 | 1.00 to 1.00 | 0.96 | 1.00 | 1.00 to 1.00 | 0.48 |
| CD3-negative lymphocytes | 1.04 | 0.99 to 1.08 | 0.10 | 1.04 | 1.00 to 1.08 | 0.08 | 1.00 | 0.97 to 1.03 | 0.85 |  | 1.00 | 0.99 to 1.02 | 0.54 | 1.01 | 0.99 to 1.02 | 0.46 | 1.01 | 0.99 to 1.02 | 0.49 |
| CD4 |  |  |  |  |  |  |  |  |  |  |  |  |  |  |  |  |  |  |  |
| Naïve T cells | 0.98 | 0.95 to 1.01 | 0.11 | 0.99 | 0.96 to 1.02 | 0.44 | 0.99 | 0.96 to 1.02 | 0.46 |  | 1.00 | 0.96 to 1.03 | 0.79 | 1.01 | 0.97 to 1.04 | 0.62 | 1.01 | 0.97 to 1.04 | 0.61 |
| Central memory T cells | 0.98 | 0.94 to 1.01 | 0.23 | 0.98 | 0.95 to 1.02 | 0.37 | 0.98 | 0.94 to 1.02 | 0.24 |  | 1.01 | 0.97 to 1.04 | 0.78 | 1.01 | 0.98 to 1.04 | 0.47 | 1.01 | 0.98 to 1.05 | 0.41 |
| Effector memory T cells | 1.04 | 1.04 to 1.04 | **<0.01** | 1.02 | 0.99 to 1.05 | 0.13 | 1.03 | 1.00 to 1.06 | 0.08 |  | 1.06 | 1.02 to 1.08 | **<0.01** | 0.92 | 0.89 to 0.94 | **<0.01** | 1.05 | 1.02 to 1.09 | **<0.01** |
| Terminally differentiated T cells | 1.12 | 0.97 to 1.29 | 0.11 | 1.09 | 0.94 to 1.26 | 0.24 | 1.06 | 0.92 to 1.21 | 0.41 |  | 1.26 | 1.00 to 1.46 | **0.02** | 1.22 | 0.96 to 1.43 | **0.04** | 1.17 | 0.93 to 1.39 | 0.10 |
| CD8 |  |  |  |  |  |  |  |  |  |  |  |  |  |  |  |  |  |  |  |
| Naïve T cells | 0.95 | 0.92 to 0.99 | **<0.01** | 0.97 | 0.93 to 1.01 | 0.20 | 0.98 | 0.94 to 1.02 | 0.26 |  | 0.92 | 0.81 to 1.01 | 0.13 | 0.98 | 0.87 to 1.05 | 0.66 | 0.98 | 0.88 to 1.04 | 0.62 |
| Central memory T cells | 0.94 | 0.83 to 1.06 | 0.33 | 0.95 | 0.83 to 1.08 | 0.43 | 0.94 | 0.82 to 1.08 | 0.39 |  | 0.96 | 0.68 to 1.22 | 0.80 | 1.01 | 0.70 to 1.28 | 0.95 | 1.03 | 0.70 to 1.34 | 0.88 |
| Effector memory T cells | 1.01 | 0.98 to 1.03 | 0.64 | 1.00 | 0.97 to 1.03 | 1.00 | 1.00 | 0.97 to 1.03 | 0.88 |  | 1.02 | 0.97 to 1.05 | 0.43 | 1.01 | 0.96 to 1.04 | 0.66 | 1.01 | 0.97 to 1.04 | 0.62 |
| Terminally differentiated T cells | 1.03 | 1.00 to 1.05 | **0.02** | 1.01 | 0.99 to 1.04 | 0.27 | 1.02 | 0.99 to 1.04 | 0.23 |  | 1.02 | 0.99 to 1.05 | 0.10 | 1.02 | 0.98 to 1.05 | 0.25 | 1.02 | 0.99 to 1.05 | 0.18 |
| Monocyte |  |  |  |  |  |  |  |  |  |  |  |  |  |  |  |  |  |  |  |
| Classical monocytes | 0.98 | 0.95 to 1.01 | 0.29 | 0.98 | 0.97 to 0.99 | **<0.01** | 0.98 | 0.94 to 1.02 | 0.26 |  | 1.00 | 0.97 to 1.02 | 0.90 | 1.00 | 0.96 to 1.02 | 0.95 | 0.99 | 0.95 to 1.02 | 0.59 |
| Intermediate monocytes | 0.95 | 0.95 to 0.95 | **<0.01** | 0.94 | 0.86 to 1.03 | 0.17 | 0.93 | 0.84 to 1.02 | 0.11 |  | 0.95 | 0.82 to 1.07 | 0.49 | 0.94 | 0.79 to 1.07 | 0.40 | 0.90 | 0.75 to 1.04 | 0.21 |
| Non-classical monocytes | 1.04 | 1.00 to 1.07 | 0.06 | 1.05 | 1.03 to 1.08 | **<0.01** | 1.06 | 1.01 to 1.12 | **0.02** |  | 1.10 | 1.01 to 1.18 | **0.02** | 1.13 | 1.03 to 1.24 | **0.01** | 1.13 | 1.01 to 1.25 | **0.03** |
|  |  |  |  |  |  |  |  |  |  |  |  |  |  |  |  |  |  |  |  |
| **Infection death** |  |  |  |  |  |  |  |  |  |  |  |  |  |  |  |  |  |  |  |
| Neutrophils | 1.01 | 0.97 to 1.05 | 0.69 | 1.01 | 0.96 to 1.05 | 0.81 | 0.99 | 0.95 to 1.04 | 0.77 |  | 1.00 | 1.00 to 1.00 | 0.69 | 1.00 | 1.00 to 1.00 | 0.51 | 1.00 | 1.00 to 1.00 | 0.39 |
| CD3-negative lymphocytes | 0.99 | 0.99 to 0.99 | **<0.01** | 0.99 | 0.95 to 1.04 | 0.81 | 1.01 | 0.98 to 1.04 | 0.61 |  | 1.00 | 0.99 to 1.01 | 0.99 | 0.99 | 0.98 to 1.01 | 0.46 | 1.00 | 0.98 to 1.01 | 0.51 |
| CD4 |  |  |  |  |  |  |  |  |  |  |  |  |  |  |  |  |  |  |  |
| Naïve T cells | 0.96 | 0.93 to 0.99 | **<0.01** | 0.97 | 0.94 to 1.00 | **0.03** | 0.98 | 0.95 to 1.01 | 0.12 |  | 0.91 | 0.86 to 0.96 | **<0.01** | 0.93 | 0.88 to 0.98 | **0.01** | 0.95 | 0.90 to 0.99 | **0.05** |
| Central memory T cells | 1.01 | 0.97 to 1.04 | 0.68 | 1.01 | 0.98 to 1.04 | 0.49 | 1.02 | 0.98 to 1.05 | 0.36 |  | 0.96 | 0.93 to 1.00 | 0.06 | 0.98 | 0.94 to 1.01 | 0.19 | 0.99 | 0.95 to 1.03 | 0.64 |
| Effector memory T cells | 1.03 | 1.03 to 1.03 | **<0.01** | 1.02 | 0.99 to 1.05 | 0.16 | 1.01 | 0.98 to 1.04 | 0.60 |  | 1.00 | 0.95 to 1.04 | 0.94 | 0.99 | 0.94 to 1.03 | 0.64 | 1.00 | 0.94 to 1.04 | 0.85 |
| Terminally differentiated T cells | 1.17 | 1.04 to 1.31 | **<0.01** | 1.11 | 0.99 to 1.24 | 0.06 | 1.09 | 0.96 to 1.20 | 0.16 |  | 1.20 | 0.94 to 1.41 | 0.08 | 1.12 | 0.88 to 1.32 | 0.23 | 1.14 | 0.90 to 1.33 | 0.17 |
| CD8 |  |  |  |  |  |  |  |  |  |  |  |  |  |  |  |  |  |  |  |
| Naïve T cells | 0.94 | 0.91 to 0.98 | **<0.01** | 0.97 | 0.93 to 1.01 | 0.10 | 0.98 | 0.94 to 1.01 | 0.24 |  | 0.79 | 0.67 to 0.90 | **<0.01** | 0.86 | 0.73 to 0.98 | 0.06 | 0.92 | 0.79 to 1.00 | 0.16 |
| Central memory T cells | 0.96 | 0.87 to 1.07 | 0.47 | 0.95 | 0.85 to 1.06 | 0.34 | 0.92 | 0.80 to 1.03 | 0.17 |  | 0.77 | 0.48 to 1.06 | 0.20 | 0.75 | 0.47 to 1.04 | 0.16 | 0.72 | 0.42 to 1.07 | 0.17 |
| Effector memory T cells | 1.02 | 1.00 to 1.05 | 0.06 | 1.02 | 1.00 to 1.04 | 0.07 | 1.02 | 1.00 to 1.04 | 0.12 |  | 1.02 | 0.99 to 1.05 | 0.18 | 1.02 | 0.98 to 1.05 | 0.21 | 1.02 | 0.99 to 1.05 | 0.18 |
| Terminally differentiated T cells | 1.02 | 1.00 to 1.04 | 0.13 | 1.00 | 0.97 to 1.02 | 0.77 | 1.00 | 0.97 to 1.02 | 0.71 |  | 1.00 | 0.96 to 1.03 | 0.93 | 0.99 | 0.94 to 1.02 | 0.48 | 0.99 | 0.95 to 1.02 | 0.58 |
| Monocyte |  |  |  |  |  |  |  |  |  |  |  |  |  |  |  |  |  |  |  |
| Classical monocytes | 1.02 | 0.98 to 1.05 | 0.34 | 1.02 | 0.98 to 1.06 | 0.29 | 1.012 | 0.98 to 1.05 | 0.42 |  | 1.01 | 0.98 to 1.02 | 0.59 | 1.00 | 0.97 to 1.02 | 0.92 | 1.00 | 0.97 to 1.02 | 0.82 |
| Intermediate monocytes | 1.02 | 0.97 to 1.07 | 0.43 | 1.01 | 0.95 to 1.08 | 0.66 | 1.01 | 0.95 to 1.07 | 0.77 |  | 1.03 | 0.92 to 1.12 | 0.59 | 0.99 | 0.88 to 1.09 | 0.92 | 0.98 | 0.87 to 1.09 | 0.77 |
| Non-classical monocytes | 0.95 | 0.91 to 1.00 | 0.06 | 0.93 | 0.88 to 0.99 | **0.02** | 0.95 | 0.89 to 1.00 | 0.07 |  | 0.93 | 0.82 to 1.03 | 0.17 | 0.88 | 0.76 to 0.99 | **0.05** | 0.88 | 0.77 to 1.01 | 0.08 |
| Univariable and multivariable Cox regression. Hazard ratios were expressed as per ten cells. Model 1 was adjusted for age, sex, and institute. Model 2 was adjusted for age, sex, institute, hemoglobin, diabetes mellitus, albumin, and C-reactive protein. | | | | | | | | | | | | | | | | | | | |

| **Supplementary Table S2.** Correlations between principal component scores, CMV IgG titers, and baseline patient characteristics | | | | | | | | | | | | |
| --- | --- | --- | --- | --- | --- | --- | --- | --- | --- | --- | --- | --- |
|  | Principal Component 1 | | | Principal Component 2 | | | Principal Component 3 | | | Log CMV IgG* | | |
|  | ρ | 95% CI | P value | ρ | 95% CI | P value | ρ | 95% CI | P value | ρ | 95% CI | P value |
| Age | 0.0016 | -0.0982 to 0.1014 | 0.9737 | 0.0719 | -0.0281 to 0.1705 | 0.1468 | 0.3523 | 0.2617 to 0.4368 | **0.0001** | 0.1156 | 0.0151 to 0.2138 | **0.0205** |
| Sex | 0.0358 | -0.0643 to 0.1351 | 0.4704 | 0.0598 | -0.0402 to 0.1587 | 0.2274 | -0.1701 | -0.2654 to -0.0715 | **0.0006** | -0.0415 | -0.1416 to 0.0594 | 0.4068 |
| Diabetes mellitus | 0.1005 | 0.0007 to 0.1983 | **0.0423** | 0.1111 | 0.0115 to 0.2086 | **0.0246** | 0.0298 | -0.0703 to 0.1292 | 0.5485 | -0.0409 | -0.1410 to 0.0601 | 0.4138 |
| Hemoglobin | 0.2080 | 0.1105 to 0.3016 | **0.0001** | 0.2155 | 0.1183 to 0.3087 | **0.0001** | -0.2163 | -0.3094 to -0.1190 | **0.0001** | -0.0994 | -0.1981 to 0.0013 | **0.0464** |
| WBC | 0.5087 | 0.4307 to 0.5791 | **0.0001** | 0.5966 | 0.5282 to 0.6573 | **0.0001** | 0.2502 | 0.1543 to 0.3415 | **0.0001** | 0.0093 | -0.0914 to 0.1099 | 0.8521 |
| Neutrophil | 0.2712 | 0.1762 to 0.3612 | **0.0001** | 0.6097 | 0.5430 to 0.6689 | **0.0001** | 0.2761 | 0.1812 to 0.3658 | **0.0001** | 0.0308 | -0.0701 to 0.1311 | 0.5382 |
| Lymphocyte | 0.9231 | 0.9068 to 0.9366 | **0.0001** | 0.1177 | 0.0181 to 0.2150 | **0.0172** | -0.0149 | -0.1146 to 0.0850 | 0.7635 | -0.0376 | -0.1378 to 0.0633 | 0.4517 |
| Monocyte | 0.5075 | 0.4294 to 0.5780 | **0.0001** | 0.6581 | 0.5975 to 0.7112 | **0.0001** | 0.2634 | 0.1680 to 0.3540 | **0.0001** | 0.0174 | -0.0834 to 0.1179 | 0.7276 |
| Albumin | 0.1509 | 0.0518 to 0.2470 | **0.0022** | 0.0464 | -0.0537 to 0.1455 | 0.3497 | -0.3325 | -0.4184 to -0.2407 | **0.0001** | -0.0905 | -0.1895 to 0.0103 | 0.0699 |
| C-reactive protein | 0.0959 | -0.1938 to 0.0040 | 0.0527 | 0.3505 | 0.2598 to 0.4351 | **0.0001** | 0.2046 | 0.1070 to 0.2983 | **0.0001** | 0.0688 | -0.0321 to 0.1683 | 0.1686 |
| Log CMV IgG* | 0.0150 | -0.0858 to 0.1155 | 0.7640 | -0.0106 | -0.1111 to 0.0902 | 0.8325 | 0.1268 | 0.0265 to 0.2246 | **0.0109** |  |  |  |
| *CMV IgG seronegative patients were excluded. Correlations between baseline characteristic parameters and the corresponding principal component scores for each subject. ρ, Spearman correlation coefficient. 95% CI, 95% confidence interval. | | | | | | | | | | | | |

| **Supplementary Table S3.** Baseline characteristics patients among PC3 quintile groups | | | | | | | |
| --- | --- | --- | --- | --- | --- | --- | --- |
|  | PC3 quintile 1 (n=82) | | PC3 quintiles 2, 3, and 4 (n=245) | | PC3 quintile 5 (n=82) | | P value |
| Age (years) | 67.87 | (12.02) | 61.42 | (11.30) | 56.14 | (12.05) | **<0.0001** |
| Sex (male%) | 45.12 |  | 45.63 |  | 68.29 |  |  |
|  |  |  |  |  |  |  |  |
| **Medical history** |  |  |  |  |  |  |  |
| Diabetes mellitus (%) | 47.56 |  | 0.4327 |  | 0.4512 |  |  |
| Hypertension (%) | 71.95 |  | 0.7551 |  | 0.8293 |  |  |
| Duration of Dialysis (years) | 5.105 | (4.252) | 6.434 | (4.915) | 6.756 | (6.172) | 0.0835 |
|  |  |  |  |  |  |  |  |
| **Laboratory data** |  |  |  |  |  |  |  |
| Hemoglobin (g/dl) | 10.57 | (1.220) | 10.83 | (1.409) | 11.44 | (1.258) | **0.0002** |
| Platelet (x 10^3^/μl) | 196.3 | (65.43) | 188.6 | (65.56) | 190 | (67.98) | 0.6868 |
| WBC (/μl) | 7270 | (2222) | 6157 | (1840) | 6288 | (1814) | **0.0009** |
| Neutrophil (/μl) | 4796 | (1979) | 4123 | (1527) | 3856 | (1367) | **0.0072** |
| Lymphocyte (/μl) | 1660 | (564.2) | 1344 | (470.8) | 1736 | (559) | **<0.0001** |
| Monocyte (/μl) | 458 | (195.8) | 372.7 | (130.6) | 392.8 | (248) | **<0.0001** |
| Total cholesterol (mg/dl) | 148.4 | (33.05) | 151.3 | (37.63) | 156.2 | (37.19) | 0.3838 |
| Triglyceride (mg/dl) | 145.3 | (89.01) | 151.4 | (101.3) | 135.6 | (81.01) | 0.5407 |
| Albumin (g/dl) | 3.804 | (0.393) | 4.036 | (0.3711) | 4.198 | (0.3099) | **<0.0001** |
| C-reactive protein (mg/dl) | 0.4500 | (0.1675-7.523) | 0.3000 | (0.1255 - 0.7525) | 0.1975 | (0.0740 - 0.5088) | **0.0006** |
| CMV IgG (U/ml) | 470.2 | (203.4-1107) | 383.9 | (178.5 - 744.5) | 336.3 | (119.4 - 607.8) | **0.0198** |
| Continuous variables are shown as mean and standard deviation. Dichotomous variables are shown as percentage. CRP and anti-CMV IgG are shown as median with interquartile range. *P* values represent comparisons between quintile groups. CMV, cytomegalovirus. To convert hemoglobin, albumin, and CRP g/dl to g/l, times 10. To convert total cholesterol mg/dl to mmol/L, times 0.0259. To convert triglyceride mg/dl to mmol/L, times 0.0113. | | | | | | | |

| **Supplementary Table S4.** Sensitivity analysis excluding covariates with high variance inflation factors (VIF > 5), for association between principal components and CMV IgG titers with all-cause mortality, cardiovascular death, and infection-related death | | | |
| --- | --- | --- | --- |
|  |  | Model 4 |  |
|  | HR | 95% CI | P value |
| **All-cause death** | | | |
| Principal Component 1 | 0.96 | 0.84 to 1.10 | 0.60 |
| Principal Component 2 | 0.85 | 0.71 to 1.01 | 0.06 |
| Principal Component 3 | 1.33 | 1.07 to 1.67 | **<0.01** |
| Log CMV IgG* | 1.29 | 0.79 to 2.10 | 0.31 |
|  | | | |
| **Cardiovascular death** | | | |
| Principal Component 1 | 1.15 | 0.93 to 1.38 | 0.19 |
| Principal Component 2 | 0.71 | 0.53 to 0.97 | **0.03** |
| Principal Component 3 | 1.48 | 1.03 to 2.14 | **0.03** |
| Log CMV IgG* | 0.94 | 0.43 to 2.06 | 0.87 |
|  | | | |
| **Infection death** | | | |
| Principal Component 1 | 0.85 | 0.67 to 1.01 | 0.16 |
| Principal Component 2 | 0.81 | 0.62 to 1.05 | 0.11 |
| Principal Component 3 | 1.33 | 0.97 to 1.84 | 0.08 |
| Log CMV IgG* | 2.08 | 0.98 to 4.49 | 0.06 |
| *CMV seronegative subjects excluded. Multivariable Cox regression. Model 4 was adjusted for age, sex, institute, diabetes mellitus, and C-reactive protein. | | | |

| **Supplementary Table S5.** Comparison of predictive models for all-cause mortality outcome | | | | | | | |
| --- | --- | --- | --- | --- | --- | --- | --- |
| Rank | Model | K | AICc | ΔAICc | Akaike weight (wᵢ) | Evidence ratio vs. clinical baseline | Evidence ratio vs. next-best model |
| 1 | PC3 immune signature | 6 | 816.2 | 0.00 | 0.553 | 11.8× | — |
| 2 | Naive CD8-only | 6 | 816.7 | 0.50 | 0.434 | 9.3× | 1.3× worse than PC3 |
| 3 | Naive CD4-only | 6 | 819.9 | 3.70 | 0.087 | 1.9× | 6.4× worse than PC3 |
| 4 | Clinical variables only (baseline) | 5 | 821.1 | 4.90 | 0.047 | 1.0× | 11.8× worse than PC3 |
| Model 4 parameters (age, sex, institute, diabetes mellitus, C-reactive protein) were included in clinical variables only model. Performance under further addition of a single immune cell subset or PC3 immune signature was compared. AICc, Akaike information criterion corrected for small sample size; ΔAICc, difference from best model; wᵢ, model probability; evidence ratios are relative likelihoods vs. the clinical baseline and vs. the top-ranked PC3 model, respectively. | | | | | | | |

**Supplementary Figure S1. Gating Strategy for Immune Cell Subsets (A)** Singlets were identified by forward scatter area and height. Lymphocytes and monocytes were gated based on forward and side scatter characteristics. For CD3-positive T lymphocytes, CD4 and CD8 T cells were separated, followed by subgrouping into naïve (CD45RA+CCR7+), central memory (CD45RA-CCR7+), effector memory (CD45RA-CCR7-), and terminally differentiated cells (CD45RA+CCR7-) for both CD4 and CD8 cells. **(B)** The CD86-positive monocytes were divided into classical monocytes (CD14++CD16-), intermediate monocytes (CD14++CD16+), and non-classical monocytes (CD14+CD16+).

**
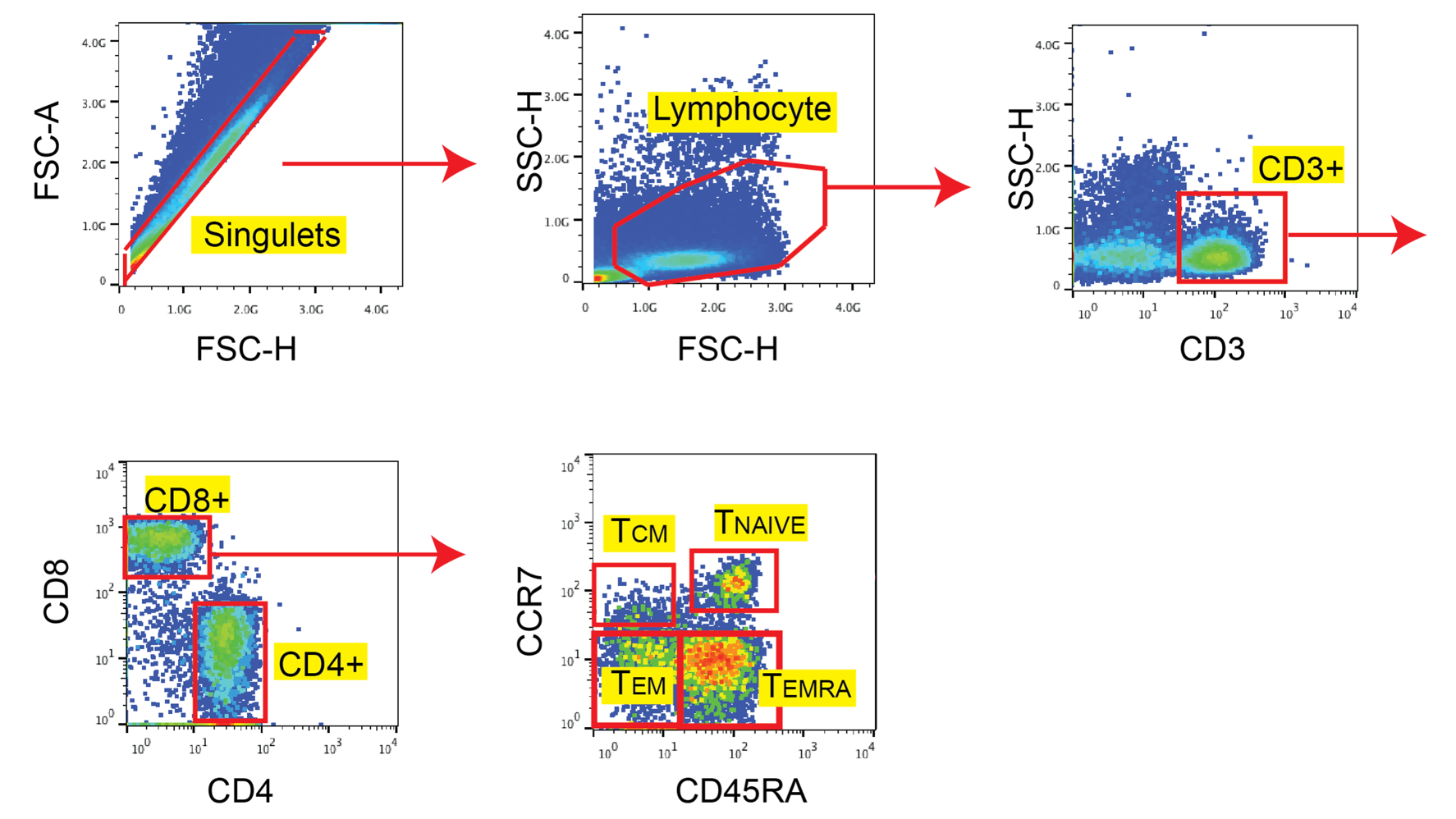
**



**
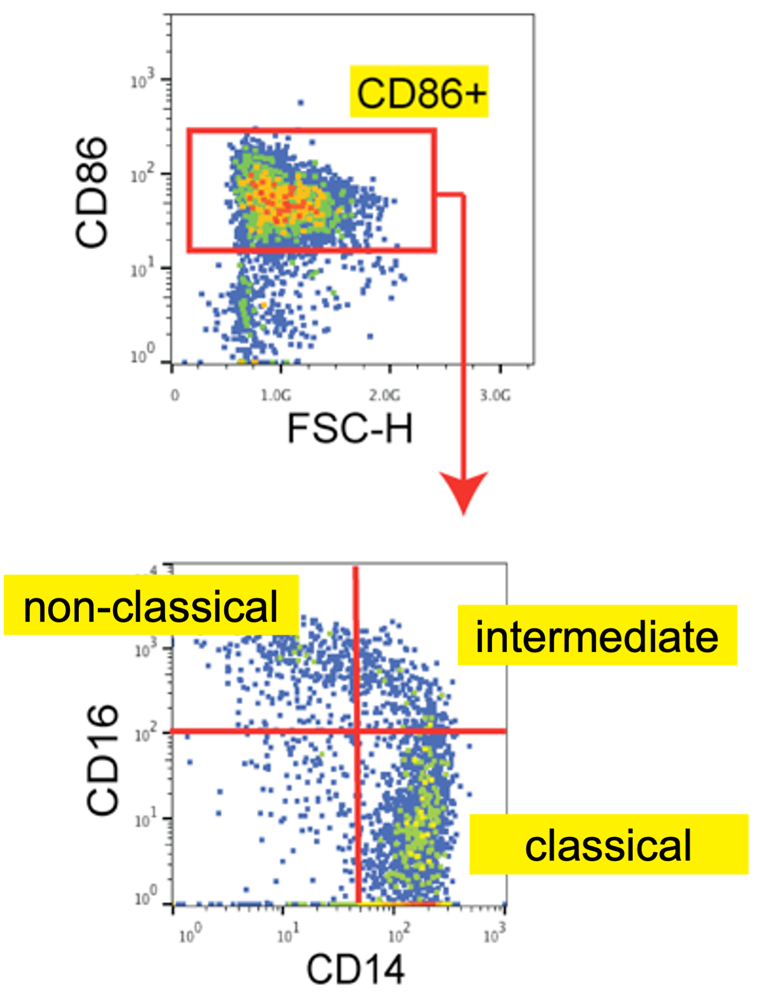
**

**Supplementary Figure S2. Characteristics of Immune Signature among Different Causes of Death.** The values of PC1, PC2, and PC3 were plotted among deceased patients with cardiovascular diseases, infectious diseases, or malignant diseases as causes of death. Cardiovascular disease as the cause of death seemed to higher scores of PC1 and PC3 compared with other causes of death, while the malignancy group exhibited a higher PC2 score. However, statistical analysis did not demonstrate difference between groups either by ANOVA or additional post-hoc analyses by Tukey's multiple comparisons test.

**Supplementary Figure S3. Immune-Adjusted Age. (A)** Simple linear regression between PC3 score and chronological age. The resulting equation defines the age-predicted PC3 score. **(B)** Relationship between chronological age and immune-adjusted age. Immune-adjusted age was defined by the hazards of death for age and PC3 score in Model 2 (Table 5). A patient with an immune-adjusted age in combination with an age-predicted PC3 score had the same risk of death as his or her chronological age in combination with his or her actual PC3 score.



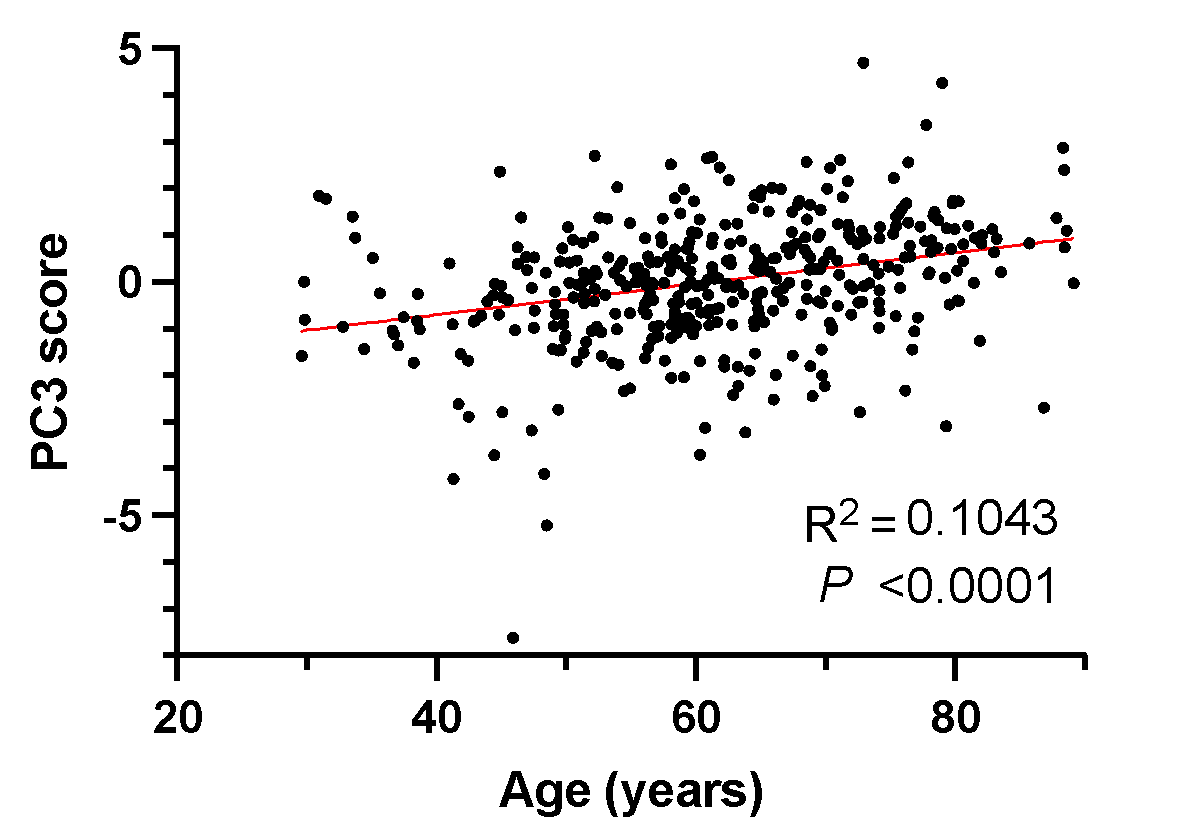


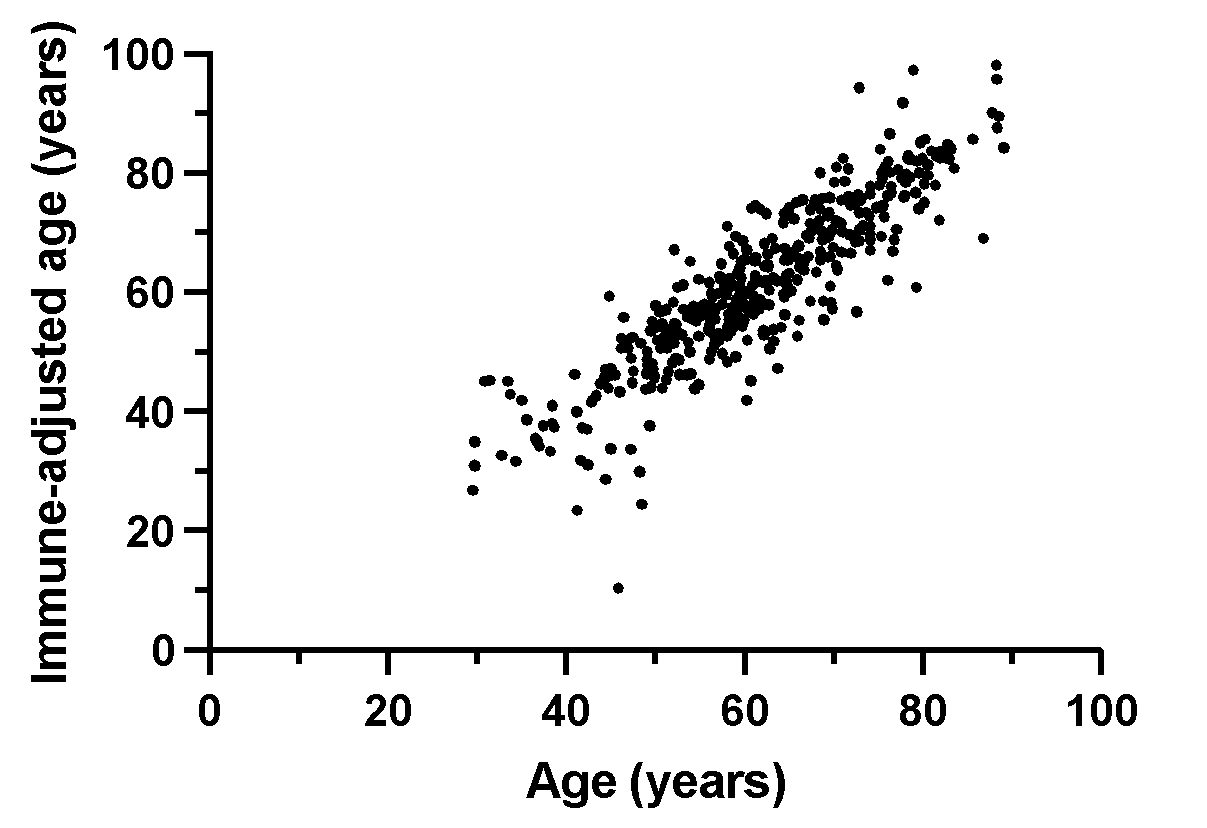

Supplement: Supplementary file 1 — Supplementary Material 1. [file 12979_2025_554_MOESM1_ESM.docx]
